# An Assessment of In-the-Wild Datasets for Multimodal Emotion Recognition

**DOI:** 10.3390/s23115184

**Published:** 2023-05-30

**Authors:** Ana Aguilera, Diego Mellado, Felipe Rojas

**Affiliations:** 1 Escuela de Ingeniería Informática, Universidad de Valparaíso, Valparaíso 2340000, Chile; 2 Doctorado en Ciencias e Ingeniería para la Salud, Universidad de Valparaíso, Valparaíso 2340000, Chile

**Keywords:** multimodal emotion recognition, in-the-wild datasets, Deep Learning

## Abstract

Multimodal emotion recognition implies the use of different resources and techniques for identifying and recognizing human emotions. A variety of data sources such as faces, speeches, voices, texts and others have to be processed simultaneously for this recognition task. However, most of the techniques, which are based mainly on Deep Learning, are trained using datasets designed and built in controlled conditions, making their applicability in real contexts with real conditions more difficult. For this reason, the aim of this work is to assess a set of in-the-wild datasets to show their strengths and weaknesses for multimodal emotion recognition. Four in-the-wild datasets are evaluated: AFEW, SFEW, MELD and AffWild2. A multimodal architecture previously designed is used to perform the evaluation and classical metrics such as accuracy and F1-Score are used to measure performance in training and to validate quantitative results. However, strengths and weaknesses of these datasets for various uses indicate that by themselves they are not appropriate for multimodal recognition due to their original purpose, e.g., face or speech recognition. Therefore, we recommend a combination of multiple datasets in order to obtain better results when new samples are being processed and a good balance in the number of samples by class.

## 1. Introduction

The ability to recognize emotions is crucial in many domains of work that use human emotional responses as a signal for marketing, technology or human–robot interaction [1]. Different models have been proposed for emotion classification or categorization [2]. The most common establish six categories such as joy, love, surprise, sadness, anger and fear [3] or a combination including disgust in place of love [4]. One of the most accepted categorizations is that of Ekman’s model. The premise of this model is that there are distinctive facial expressions. Their labels have widely been used by most of the published facial inference research studies over the last 50 years [5]. Some authors have also included the neutral emotion [6,7].

Automatic emotion recognition (ER) is a topic of study that has attracted a great deal of interest. This consists in practice of identifying human emotion from signals such as facial expression, speech and text [8]. A variety of information sources is usually considered since people naturally express emotions in simultaneous different ways, such as facial gestures [9] or other types of gestures with the hands or arms [10], or posture [11], all related to the environment of the interaction [12]. When different modalities for ER are used, the processing is known as multimodal ER [13]. Because each source by itself can produce an ER, the fusion of these results could mitigate present limitations in some single-source approaches, thus obtaining more accurate detection [14].

Deep architectures and learning techniques have shown effectiveness for multimodal ER [15,16,17] and sentiment analysis [18] with classification tasks. Effectively, Deep Learning supports customizable structures for ER, allowing high-level data abstraction. In Deep Learning techniques, Deep Neural Networks are employed to collect distinguishing traits from high-level data representation [19]. The most common techniques include Long Short-Term Memory (LSTM) [13,20], Convolutional Neural Networks [21,22], fully connected Multi-Layer Perceptrons (MLP) [23], Recurrent Neural Networks (RNN) [24], autoencoders [25] and Convolutional Deep Belief Network [26].

These architectures have been trained for ER using multimodal signals such as facial and audio gestures, audio and written language, physiological signals and different variations in those modalities [27]. Some works are focused on images (face, pose), audio (speech) and text modalities fused with a fusion method [20,23,28].

Other works consider physiological signals [21,22,24,25] and yet others include combinations of all these modalities [26]. Deep Learning techniques have progressed rapidly in computer vision applications to address detection, localization, estimation and classification issues [29]. An embedding-based Deep Learning approach for 3D cell instance segmentation and tracking to learn spatial, temporal and 3D context information simultaneously was introduced by [30]. This method was also used for face recognition in [31] using a pseudo RGB-D (Red, Green, Blue—Depth) framework and providing data-driven ways to generate depth maps from 2D face images. The problem of extracting and curating individual subplots from compound figures was addressed in [32] with a simple compound figure separation framework that uses weak classification annotations from individual images. Deep transfer learning from face recognition methods were developed in order to explore disease identification from uncontrolled 2D face images [33]. Deep Learning has been successfully combined with data augmentation techniques for introducing different intensities of interference to the spectrum of radio signals [34].

As shown, multimodal ER is becoming more popular in the affective computing research community in order to overcome the constraints imposed by processing just one type of data and to improve recognition robustness [22]. However, despite the progress of ER using Deep Learning techniques that is shown in a large number of studies, most of them use datasets built in laboratory environments, such as IEMOCAP [20,23], AMIGOS [24], RECOLA [28], DEAP [21], SEED, SEED-IV, SEED-V, DEAP and DREAMER [25], etc. The fundamental problem with any recognition system is the lack of data or the training with real data which may affect its generalization to examples that have not been seen during the training process [21,35]. Furthermore, the datasets used for training of ER models have been designed in controlled laboratory environments and are significantly different from what happens in real conditions in terms of brightness, noise level, etc. [36]. Most existing methods have shown good recognition accuracy on lab-controlled datasets, but they deliver much lower accuracy in a real-world uncontrolled environments. Compared to these, datasets from non-controlled environments are also referred to as “in-the-wild” data [37]. Machine learning communities have accepted that progress in a particular application domain is considerably accelerated when a large number of datasets are collected in unconstrained conditions [38,39]. With such data, multimodal analysis could focus on spontaneous behaviors and on behaviors captured in unconstrained conditions [40].

Therefore, the aim of this work is to assess a set of in-the-wild datasets in order to show their strengths and weaknesses for multimodal emotion recognition. Four in-the-wild datasets are to be evaluated: AFEW, SFEW, MELD and AffWild2. The main contributions of this work are as follows:

- A review of different works that use in-the-wild datasets. This review comprises works from recent years concerning multimodal emotion recognition methods and the datasets used in their experiments. A detailed description of these datasets is also provided.
- A descriptive analysis of the four selected datasets for our study. This description includes the frequency distribution of emotions, visualization of some samples and details related to the original extraction sources.
- An evaluation in terms of performance using an ensemble of Deep Learning architectures and fusion methods. The tests include ablation experiments reporting individual results by modality and fusion results.

This work is structured in several sections. Section 2 presents a review of different studies concerning emotion recognition using in-the-wild datasets. Section 3 presents descriptions of the selected in-the-wild datasets. Section 4 provides a brief description of the ensemble of architectures with which the datasets were evaluated. Section 5 presents results of the training process with the in-the-wild datasets using the ensemble of Deep Learning architectures. Section 6 discusses the main results and recommendations and finally Section 7 presents conclusions and future works.

## 2. Related Work

Affective computing is mainly a data-driven research area underpinned by self-built or public databases and datasets. Most of the available data have been collected and stored in controlled conditions in labs. However, there are few existing multimodal emotion databases collected in real-world conditions and those that exist are small and are typically made with a limited number of subjects and expressed in a single language [41]. This review of works focuses on some of these last datasets collected under in-the-wild conditions.

Some projects developed in the context of facial emotion recognition [42] and multi-modal approaches have used or created their own datasets for experimentation. Riaz et al. [43] conducted tests on Facial Expression Recognition 2013 (FER-2013), the Extended Cohn-Kanade Dataset (CK+) and the Real-world Affective Faces Database (RAF-DB) benchmark datasets. The implementation, called by the authors eXnet (Expression Net), consisted of a Convolutional Neural Network (CNN) architecture based on parallel feature extraction for facial emotion recognition (FER) in the wild. This method achieved higher accuracy on the studied datasets (71.67% for FER-2013, 95.63% for CK+ and 84% for RAF-DB), while using a smaller number of parameters and smaller size on disk than other methods. Chen et al. [41] proposed the Multi-Modal Attention module (MMA) to fuse multi-modal features adaptively on the HEU-part1 and HEU-part2 Emotion databases. The HEU Egm motion database contains a total of 19,004 video clips tagged in 10 emotions (anger, bored, confused, disappointed, disgust, fear, happy, neutral, sad, surprise). For extracting raw features of face, body and audio, CNN+GRU, 3D ResNeXt and OpenSMILE were used and combined with the MMA. Comparative tests were performed on the AFEW and CK+ Datasets. The best results using all modalities and the proposed MMA were 49.22% for HEU-part1 and 55.04% for HEU-part2 with the validation sets.

The Emotion Recognition in the Wild (EmotiW) challenge is a benchmarking effort run as a grand challenge of the several International Conferences on Multimodal Interaction. Various editions starting in 2012 involve different tasks for ER in the wild [44]. Various research projects in affective computing, computer vision, speech processing and machine learning have been developed. Some of the best works presented in these areas are summarized below. Samadiani et al. [36] proposed a video ER that combines visual and audio features on the Acted Facial Expression in the Wild (AFEW) 4.0 dataset. The authors considered the head pose challenge in the videos captured in the wild and proposed a feature-extraction method to handle it. A sparse kernel representation was applied to concatenate the features and a joint sparsity concentration index measurement was used as the decision strategy to indicate the effectiveness of modalities. A Random Forest classifier was used to classify seven basic emotions (angry, disgust, fear, happy, neutral, sad and surprise), achieving an accuracy of 39.71%. Hu et al. [45] experimented in the EmotiW 2017 audio–video-based ER sub-challenge with AFEW 7.0. For this, the authors presented a supervised scoring ensemble that provides dense supervision in diverse feature layers of a deep CNN and bridges class-wise scoring activations for second-level supervision. It extended the idea of deep supervision by adding supervision to deep, intermediate and shallow layers. Afterwards, a fusion structure concatenated class-wise scoring activations from diverse complementary feature layers. The results showed the best accuracy of 60.34% on the ER task for this sub-challenge. Li et al. [46] proposed a framework for video-based ER in the wild taking visual information from facial expression sequences and speech information from audio. This was done in the context of the EmotiW 2019 audio–video-based ER sub-challenge with AFEW 7.0. Different deep networks were used (VGG-Face, BLSTM, ResNet-18, DenseNet-121, VGG-AffectNet) and a fusion method based on weighted sum was used to combine the outputs of the networks. Additionally, to take advantage of the facial expression information, the VGG16 network was trained on the AffectNet dataset to learn a specialized facial expression recognition model. The best results show an accuracy of 62.78% after the fusion method. Salah et al. [47] proposed an approach for video-based ER in the wild using deep transfer learning and score fusion in the context of EmotiW Challenge 2015 and 2016. For the visual modality, this approach used summarizing functionals of complementary visual descriptors, and for the audio modality a standard computational pipeline for paralinguistics was proposed. Both audio and visual features were combined with least squares regression-based classifiers and weighted score-level fusion. The datasets used corresponded to the AFEW corpus and FER-2013. This approach achieved accuracies of 54.55% and 52.11%

Some researches on the sub-challenge of the ER in-the-Wild Challenge (EmotiW) have focused on the automatic classification of a set of static images into seven basic emotions on the SFEW (Static Facial Expression in Wild) dataset. SFEW is a static subset of AFEW that addresses recognizing more spontaneous facial expressions. Yu and Zhang [48] proposed a method with a face-detection module based on an ensemble of three face detectors, followed by a classification module with an ensemble of multiple deep convolutional neural networks. The three detectors were joint cascade detection and alignment (JDA), Deep-CNN-based (DCNN) and mixtures of trees (MoT). Pre-trained models were executed on a larger dataset provided by the Facial Expression Recognition (FER) Challenge 2013 and afterwards fine-tuned on the training set of SFEW 2.0. This approach achieved 55.96% and 61.29%, respectively, on the validation and test set of SFEW 2.0, surpassing the challenge baseline of 35.96% and 39.13% with significant gains. Munir et al. [49] proposed a technique composed of three major modules, (a) preprocessing with Fast Fourier Transform (FFT) and Contrast Limited Adaptive Histogram Equalization (CLAHE) methods, (b) generation of merged binary pattern code (MBPC) by pixel, (c) dimensionality reduction with a Principal Component Analysis (PCA) and a classifier to identify the expression. The SFEW dataset was selected for experimentation. Results show 96.5% and 67.2% accuracy for the holistic and division-based approaches, respectively. Cai et al. [50] designed a novel island loss (IL-CNN) to simultaneously increase inter-class separability and intra-class compactness. The proposed method included three convolutional layers, each of which was followed by a PReLU layer and a batch normalization layer (BN). A maximum grouping layer was used after each of the first two BN layers. After the third convolutional layer, two fully connected layers (FC) and one Softmax DRMF were included. Ruan et al. [51] proposed an ensemble of networks for facial ER, composed of four parts: (a) the backbone network (ResNet-18) that extracts basic CNN features; (b) a Feature Decomposition Network (FDN) that decomposes the basic feature into a set of facial action-aware latent features; (c) a Feature Reconstruction Network (FRN) that learns an intra-feature relation weight and an inter-feature relation weight for each latent feature and reconstructs the expression feature; the FRN contains two modules: an Intra-feature Relation Modeling module (Intra-RM) and an Inter-feature Relation Modeling module (Inter-RM); (d) an Expression Prediction Network (EPN) that predicts an expression label. Experimental results were obtained on both the in-the-lab databases (including CK+, MMI and Oulu-CASIA) and the in-the-wild databases (including RAF-DB and SFEW). The accuracy results on RAF-DB and SFEW were 89.47% and 62.16%, respectively.

Emotion recognition in conversations (ERC) is another challenging task that has recently gained interest due to its potential applications. Conversation in its natural form is multimodal. ERC presents several challenges such as conversational context modeling, emotion shift of the interlocutors and others that make the task more difficult to address [52]. Xie et al. [53] proposed a multimodal emotion classification architecture on the Multimodal Emotion Lines Dataset (MELD), including three modalities (textual, audio and face). Three separate prediction models were trained by Generative Pre-trained Transformer (GPT) for text, WaveRNN for audio and FaceNet+GRU for images. A transformer-based fusion mechanism with Embracenet was used to provide multimodal feature fusion. The architecture considered both the joint relations among the modalities and fused different sources of the representation vector. The results showed an accuracy of 65.0% and an F1-Score of 64.0%. Ho et al. [54] presented a multimodal approach for speech emotion recognition based on a Multi-Level Multi-Head Fusion Attention mechanism and a recurrent neural network (RNN) with two modalities (audio and text). The mel-frequency cepstrum (MFCC) from raw signals using the OpenSMILE toolbox was used to determine audio features. A pre-trained model of bidirectional encoder representations from transformers (BERT) was used for embedding text information. A multi-head attention technique fused all feature representations. The experimentation was performed on three databases, Interactive Emotional Motion Capture (IEMOCAP), MELD and CMU Multimodal Opinion Sentiment and Emotion Intensity (CMU-MOSEI). The results showed an accuracy of 63.26% and an F1-Score of 60.59%. Hu et al. [55] introduced a multimodal fused graph convolutional network (MMGCN) for ERC utilizing both multimodal and long-distance contextual information. The MMGCN consisted of three key components: Modality Encoder, Multimodal Graph Convolutional Network and Emotion Classifier. Experiments were conduced on two public benchmark datasets, IEMOCAP and MELD. The results showed accuracy of 66.22% and 58.65% for IEMOCAP and MELD, respectively.

The First Affect-in-the-wild Challenge (AffWild Challenge) was organized in conjunction with the Computer Vision and Pattern Recognition Conference (CVPR) 2017, using the AffWild database [56]. Later this database was extended with 260 more subjects and 1,413,000 new video frames, after which it was called AffWild2 [57]. Some researchers have since experimented with AffWild2. Barros and Sciutti [58] implemented a Deep Neural Network based on VGG16, with a final feature layer that classifies arousal, valence and emotion categories. This was expanded to also process sequential data and achieved an F1-Score of 0.38. Liu et al. [59] proposed a framework that extracts facial features and applies regularization in order to focus on more evident samples, while more uncertain expressions are relabeled. This framework used ResNet-18 [60] and DenseNet [61] architectures, pretrained with the MS-Celeb-1M dataset [62], and achieved a mean accuracy of 62.78%. Yu et al. [63] proposed an ensemble learning approach, using different architectures such as ResNet, EfficientNet [64] and InceptionNet [65] and training with multiple folds between the data in order to enhance their performance. This achieved a mean F1-Score of 0.255. Zhang et al. [66] proposed a unified transformer-based multimodal framework for Action Unit detection and also expression recognition. This framework is made of three gated recurrent neural networks (GRU) and a multilayer perceptron (MLP). A transformer-based fusion module was used to integrate the static vision features and the dynamic multimodal features. The expression F1-Scores of models that are trained and tested on six different folds were calculated (including the original training/validation set of the AffWild2 dataset). The results showed mean F1-Scores of 39.4, 37.9, 41.1, 37.8, 37.3 and 36.1, respectively, for each fold.

Table 1 summarizes works presented in this section. Table 2 summarizes datasets or databases used in these works.

## 3. A Selection of In-the-Wild Datasets

We selected four in-the-wild datasets for testing: AFEW [68], SFEW [68], MELD [52] and AffWild2 [57]. These datasets were selected primarily because of their accessibility. Requests for access were made directly to their authors and access was granted for academic purposes. A description of each dataset follows.

**Figure 1:**
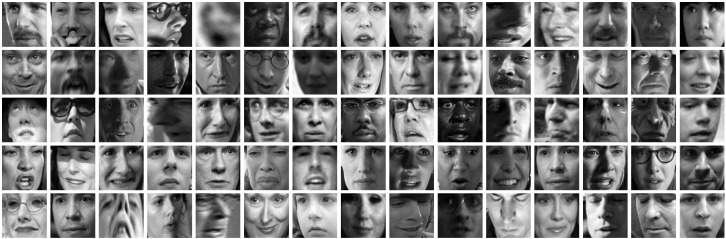
Center-faced crops present in AFEW dataset.

**Figure 2:**
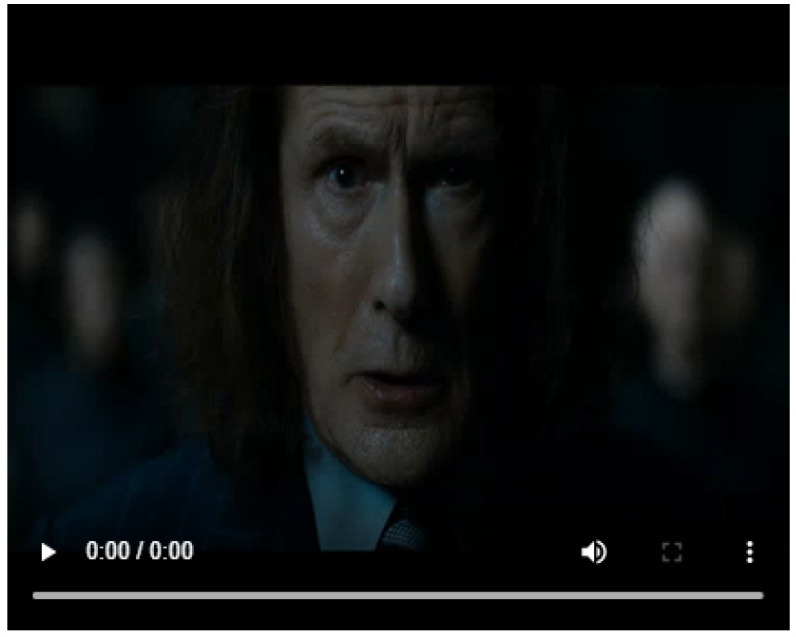
Anger emotion video.

**Figure 3:**
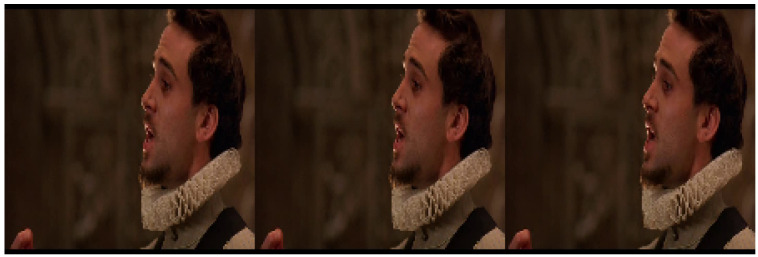
Frames in a video.

**Figure 4:**
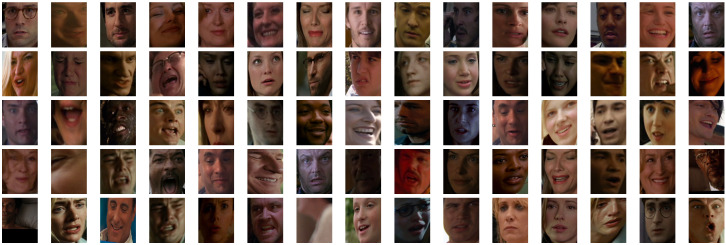
Crops of faces from SFEW dataset.

**Figure 5:**
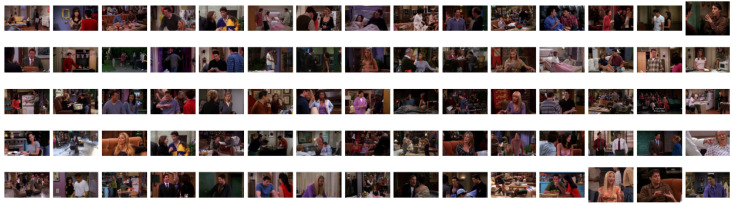
Raw frames from MELD dataset.

**Figure 6:**
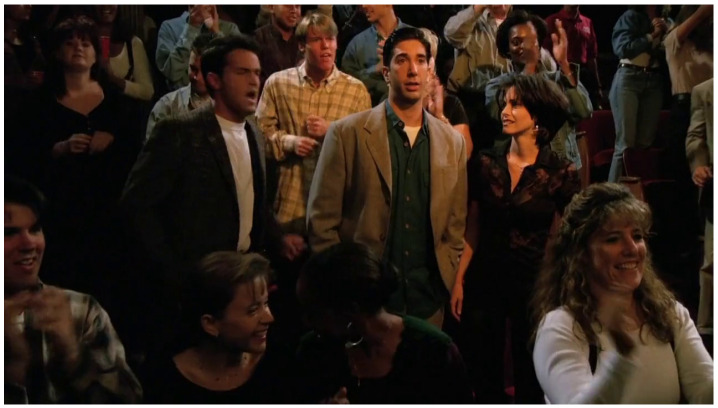
Specific frame from MELD dataset.

**Figure 7:**
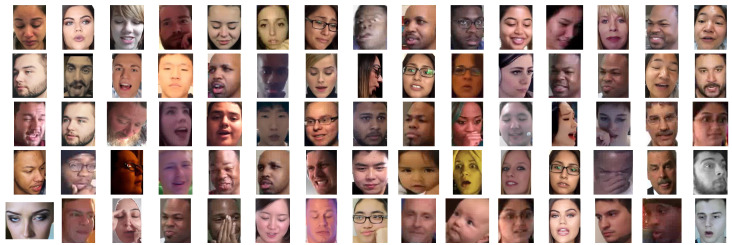
Examples of cropped faces in AffWild2.

- AFEW: This is a dyna.mic temporal facial expressions data corpus proposed by A. Dhall et al. in 2011 [77]. It has been used as a database for the Emotion Recognition in-the-wild Challenge (EmotiW) since 2013. Different versions have appeared every year for each challenge. For the sake of simplicity, the following refers to the most recent version as AFEW. It consists of close to real world environment instances, extracted from movies and reality TV shows, including 1809 video clips of 300–5400 ms with various head poses, occlusions and lighting. The database contains a large age range of subjects from 1 to 70 years from various races, genders and ages and with multiple subjects in a scene. Around 330 subjects have been labeled with information such as name, age of character, age of actor, gender, pose and individual facial expressions. AFEW consists of separate training (773), validation (383) and test (653) video clips, in which samples are tagged with discrete emotion labels: the six universal emotions (angry, disgust, fear, happy, sad and surprise) and neutral. Audio and video are in WAV and AVI formats. Modalities to explore in this database include facial, audio and posture. Figure 1 shows some samples from this dataset.
  Each emotion is represented by a set of short videos with different actors showing the emotion in different situations, e.g., a person with a face showing anger (Figure 2). It should be noted that the test folder does not contain the videos categorized by emotion and that the quality of the videos is variable, with some containing scenes with different levels of image quality (Figure 3). The official site of this dataset is the School of Computing, Australian National University (https://cs.anu.edu.au/few/, accessed on 27 May 2023).
- SFEW: A static facial expression database in the wild (SFEW) was created with frames containing facial expressions extracted from AFEW. The database includes unconstrained facial expressions, varied head poses, a large age range, occlusions, varied focus, different resolutions of face and close to real world illumination. Frames extracted were labeled with two independent labelers based on the label of the sequence in AFEW with six basic expressions: angry, disgust, fear, happy, sad, surprise and neutral. It is composed of 1766 images, including 958 for training, 436 for validation and 372 for testing. Each of the images has been assigned to one of seven expression categories, neutral and the six basic expressions. The expression labels of the training and validation sets are provided, while those of the testing set are held back by the challenge organizers. [73]. Figure 4 shows some samples of this database. This corpus is a widely used benchmark database for facial expression recognition in the wild. The official website is the same as AFEW. Table 3 shows the number of images available in SFEW per emotion.
- MELD: the Multimodal EmotionLines Dataset (MELD) is an extension and enhancement of EmotionLines [78]. The MELD corpus was constructed by extracting the starting and ending timestamps of all utterances from every dialog in the EmotionLines dataset, given that timestamps of the utterances in a dialog must be in an increasing order and all the utterances in a dialog have to belong to the same episode and scene. After obtaining the timestamp of each utterance, the corresponding audiovisual clips were extracted from the source episode followed by the extraction of audio content from these clips. The audio files were formatted as 16-bit PCM WAV files. The final dataset includes visual, audio and textual modalities for each utterance. MELD contains about 13,000 utterances from 1433 dialogs from the TV series Friends with different speakers participating in these dialogs. It provides multimodal sources including not only textual dialogs, but also their corresponding visual and audio counterparts. Each utterance is annotated with emotion and sentiment labels. Emotions correspond to Ekman’s six universal emotions (joy, sadness, fear, anger, surprise and disgust) with an additional emotion label neutral. For sentiments, three classes were distinguished as negative, positive and neutral [52]. Figure 5 shows an extract of this dataset. Each frame is an extract collected from a video and most of these frames contain several people expressing different emotions (Figure 6). The dataset contains only the raw data for the complete video frames; individual faces are not cropped or tagged.
  Within the MELD site (https://github.com/declare-lab/MELD, accessed on 27 May 2023), the data are structured in different folders. In the data folder in particular, there are three different CSV files which corresponds to train, dev and test datasets with 9990, 1110 and 2611 lines, respectively. Each file has the same structure, containing utterance, speaker, emotion and sentiment information, as well as data concerning the source.
- AffWild2: This is an extension of the AffWild dataset which was designed for the First Affect-in-the-Wild Challenge [79]. AffWild collected data available in video-sharing websites such as YouTube and selected videos that display the affective behavior of people, for example, videos that display the behavior of people when watching a trailer, a movie, a disturbing clip or reactions to pranks. It was designed to train and test an end-to-end deep neural architecture for the estimation of continuous emotion dimensions based on visual cues [56] and was annotated in terms of the valence-arousal dimensions. A total of 298 videos displaying reactions of 200 subjects, with a total video duration of more than 30 h, were collected. Later, AffWild2 extended the data with 260 more subjects and 1,413,000 new video frames [57]. The videos were downloaded from YouTube and have large variations in pose, age, illumination conditions, ethnicity and profession. The set contains 558 videos with 2.8 million frames in total of people reacting to events or audiovisual content or speaking with the camera. The videos involve a wide range in subjects’ age, ethnicity and profession and they have different head poses, illumination conditions, occlusions and emotions. The videos were processed, trimmed and reformatted to MP4. Frames were annotated for three tasks (valence arousal, emotion classification and action unit detection) [80]. This dataset is also distributed as a set of cropped frames from each video, centered on the face. The number of images available is shown in Table 4.
  For emotion classification, each frame was annotated with one of the six main emotions (anger, disgust, fear, happiness, sadness, surprise, other) and a neutral label. Some frames are labeled as discarded (with a “−1”) for this task. The official site of AffWild2 belongs to the Intelligent Behaviour Understanding Group (iBUG), Department of Computing at Imperial College London (https://ibug.doc.ic.ac.uk/resources/aff-wild2/, accessed on 27 May 2023). Figure 7 shows some samples from the AffWild2 dataset.

Table 5 summarizes some insights of these in-the-wild datasets.

### 3.1. Preprocessing

In this section, we describe the steps used to preprocess the four datasets by modality group.

#### 3.1.1. Face Modality

For this modality, AFEW, SFEW and AffWild2 had available cropped and centered segments of each video frame with a labeled face of the subject on screen. Figure 1, Figure 4 and Figure 7 show some frames extracted from their original video source with a face detection algorithm.

**Figure 8:**
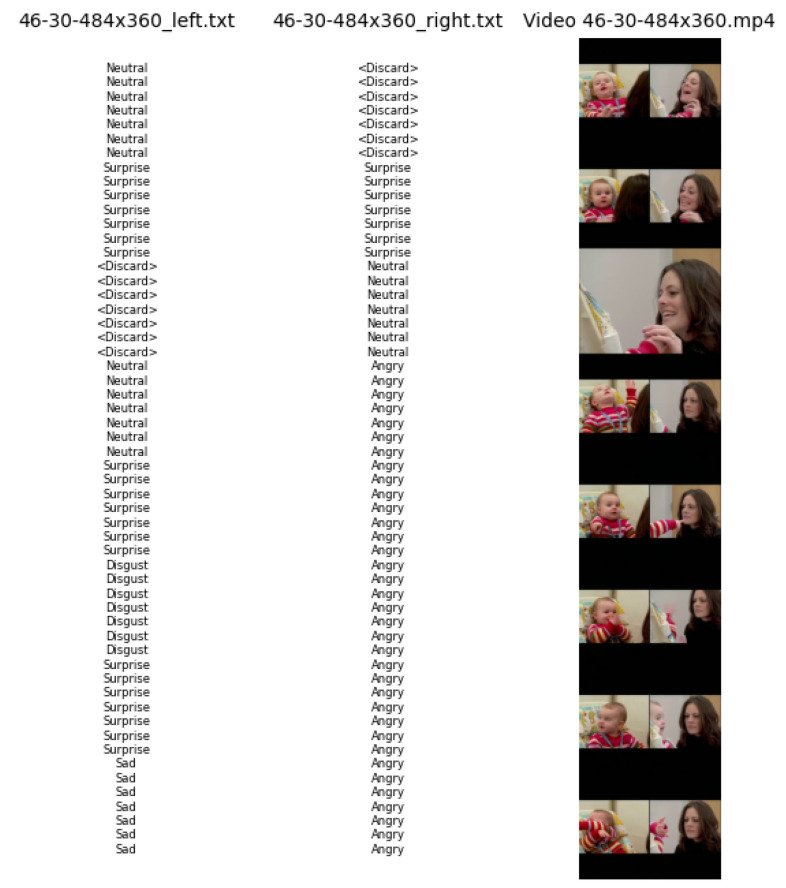
Example of two annotation files from the same video, showing both participants and the label at the current frame of video.

Because AFEW and SFEW were distributed among various folders by emotion, we created a CSV file containing the path of each frame and its label. In the AffWild2 dataset, each video has a corresponding annotation file. A line in these files indicates the label for each frame. Few of these videos contain more than one person in focus. In these cases, the videos have several annotation files (one per person), each with a label corresponding to the person it refers to. Figure 8 shows an example of this. All frames missing a category label were annotated with −1, indicating the frame should be discarded.

**Figure 9:**
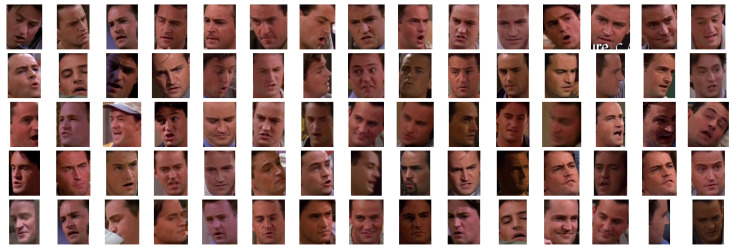
Examples of cropped faces in MELD.

MELD does not provide cropped images per character, since this dataset is focused on audio and text classifications. Many samples contain more than one person by frame and so we extracted from each video the cropped faces of each character. Afterwards, the frames for each character were matched with the corresponding labeled dialog in order to tag these frames. We subsampled from each video a small number of frames, obtaining around 3711 images in order to fine-tune a VGGFace architecture to identify each subject (characters from the sitcom Friends) from these crops. This classifier achieved an accuracy of 99.87%. Finally, we matched each character from the video to their labeled emotion, selecting the best identified character from each sample, based on a classifier probability threshold of over 70% certainty in its prediction. Then, we saved these samples in a folder with each image being identified via its filename, following the format result_dia[ID]_utt[ID].[n].png, where dia and utt correspond to the dialog and utterance number on the dataset label’s csv file, ID being the ID code from the corresponding video and *n* the number of frames of the specific character from this video. The result of this cropping process is shown in Figure 9.

To generate a diverse set of images for training, we applied some random augmentation operations to the training data. These operations imply transformations, which had a 50% chance of being applied, modifying an image. Transformations include flipping, rotation in the range [−10,10] degrees, random contrast and brightness or addition of Poissonnoise; all of them are applied independently.

As a consequence of the VGG model used for this modality, which requires a defined input size, we had to resize the original images (128×128 pixels in AFEW, 181×143 pixels in SFEW and 246±176×128±129 pixels in AffWild2) to 48×48 pixels, using a bilinear interpolation function provided by the scikit-image library in Python.

#### 3.1.2. Audio Modality

For the audio data, we first extracted the audio of each original video at a 22.4 kHz sample rate using ffmpeg [81] on all datasets.

**Figure 10:**
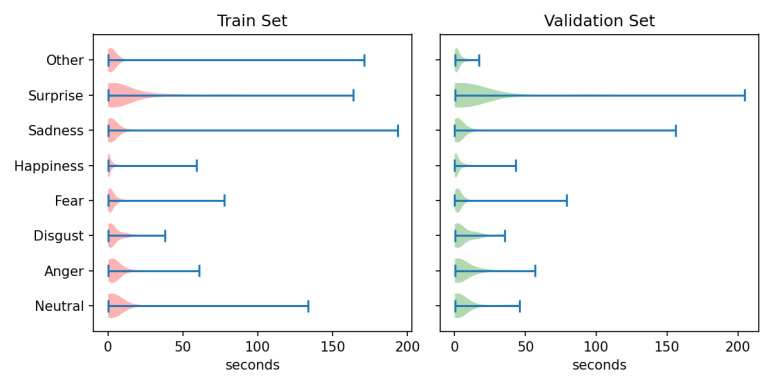
Duration of segments for each emotion in train and validation set of AffWild2.

In particular, each video in AffWild2 contains a number of labeled emotion utterances, which define video segments. Audio clips were extracted from each of these segments using Librosa [82] and were labeled with the same emotion as their corresponding utterance. These segments have a mean duration of 3.88±10.18 s, with lengths between ∼30 ms (a frame of video) and 200 s of duration. Figure 10 shows the length distribution for each emotion on training and validation sets. On other datasets, each video sample represents a single utterance; therefore, audio can be used directly from each sample. Each audio item has a duration of up to 6.8 s (average 2.458±1.017 s).

**Figure 11:**
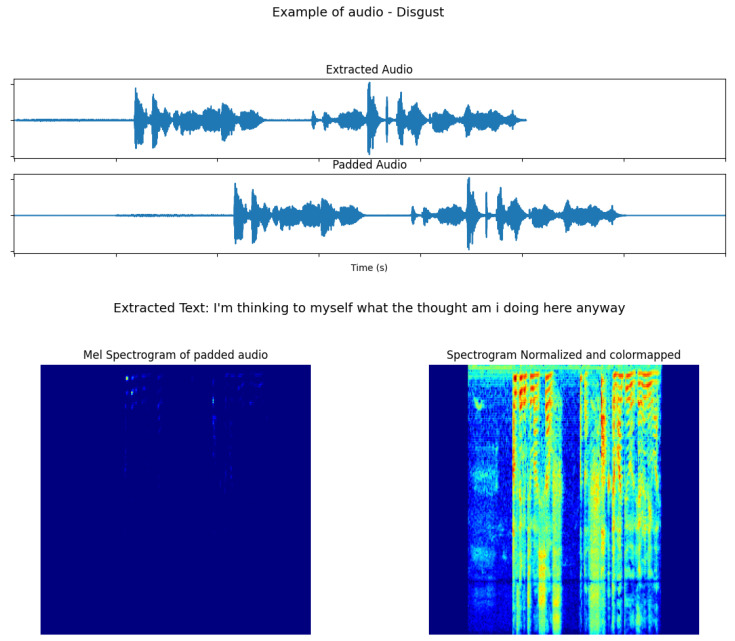
Visualization of one audio sample and its preprocessing for our audio model.

On all datasets, we set the input duration for our model to 7 seconds. Shorter segments were padded with zeros and centered (second plot in Figure 11), whereas larger segments were cut to 7 seconds from a random starting position. Afterwards, the segments were converted to Mel Spectrograms using Librosa (bottom-left image in Figure 11), the scale was transformed to decibels (using Librosa.power_to_db()) and finally it was normalized to the range [0–1] and colormapped to encode amplitude information as color in an image, as suggested in the work of Lech et al. [83]. Figure 11 shows this process.

For training, each audio sample was augmented with random displacement from the center of the padded audio and the addition of Gaussian noise with $\sigma =1\times 10^{-5}$.

#### 3.1.3. Text Modality

For the text model, we used the same audio segments that were previously extracted from the videos, without the duration normalization and converted them to text using the Speechbrain [84] EncoderDecoderASR Transformer model. An example of the extracted text is shown in Figure 11. For the MELD dataset, a transcription of the audio of each video sample is provided within the data. Thus, we used this transcription as input for our text model.

No data augmentation was applied to text mode data.

## 4. A Multimodal Framework for Emotion Recognition

**Figure 12:**
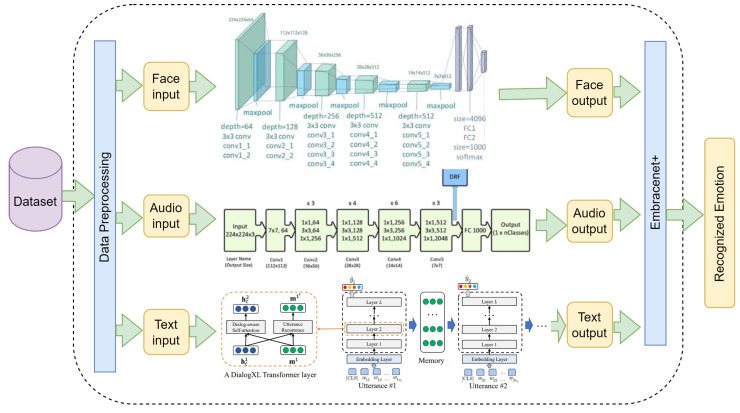
Multimodal Emotion Recognition Architecture proposed by [15], modified with a ResNet50 as the audio classifier.

**Figure 13:**
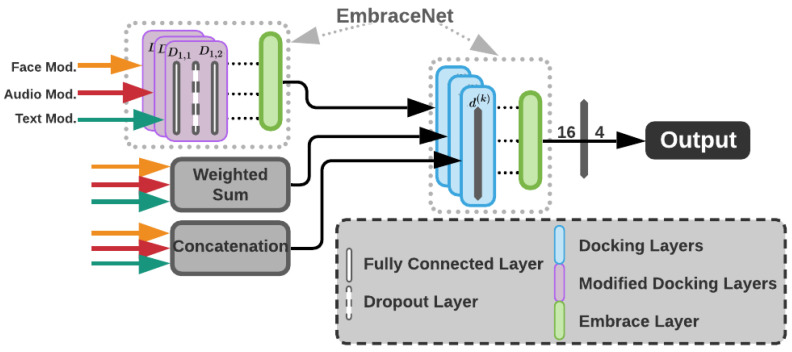
Embracenet+ Architecture proposed by [15].

For assessment of the in-the-wild datasets mentioned in Section 3, we employed an architecture designed and implemented in a previous work [15] with minor modifications, detailed below. To support emotion recognition using different modalities, specifically face, audio and text, a multimodal architecture is required. This architecture is made up of three individual components: a face component based on a VGG19 network, an audio component based on ResNet50 and a text component based on XLnet called DialogXL [85]. Each component individually recognizes an emotion (modality output). Afterwards, all of these outputs are fused, producing the final recognized emotion. Figure 12 shows the configuration of this architecture. The details are summarized below:

- Face Modality processing. We used a VGG19 architecture [86] as our classifier. This model is built using 19 convolutional layers with filters of size 3×3. Following 2 layers with 64 channels each, the output is reduced using a max pooling operation of size 2×2. This continues with alternation of pooling with groups of 2 layers of 128 channels, 4 layers of 256 channels, 4 layers of 512 channels and 4 layers of 512 channels. After a final max pooling operation, the output goes to an MLP network with 3 dense layers of sizes 4096, 4096 and 1000 and then a final layer with a Softmax activation function.
- Audio Modality processing. We used a ResNet50 architecture [60] trained from scratch, replacing the original architecture proposed by Venkataramanan and Rajamohan [87], which was used on our previous work. Our expected input is an image with size 224×224, representing the spectrogram of the input audio sample. After a convolutional layer with filter size of 7×7 and 64 channels, the input is passed through a number of residual blocks. These residual blocks are composed of three convolutional layers of filter sizes 1×1, 3×3 and 1×1, then the input of the block is added to the block output, providing residual information of higher level features. After a number of groups, the output is max pooled, reducing its size. On ResNet50, this operation occurs after 3, 4, 6 and 3 ResNet blocks. Finally, the output is average pooled, creating a 2048-length vector of features. This vector is then passed to a dense layer and an output layer with a Softmax activation. This last output layer has a number of neurons corresponding to the number of emotions. This is shown in the audio segment of Figure 12.
- Text Modality processing. We used DialogXL [85], a PyTorch implementation for Emotion Recognition in Conversation (ERC) based on XLNet. It consists of an embedding layer, 12 Transformer layers and a feed-forward neural network. DialogXL has an enhanced memory to store longer historical context and a Dialog-Aware Self-Attention component to deal with the multi-party structures. The recurrence mechanism of XLNet was modified from segment-level to utterance-level in order to better model the conversational data. Additionally, Dialog-Aware Self-Attention was used in replacement of the vanilla self-attention in XLNet to capture useful intra- and interspeaker dependencies. Every utterance (sentence) made by a speaker is routed via an embedding layer, which tokenizes the sentence into a series of vectors. This representation is then fed into a stack of neural networks, each layer of which outputs a vector that is fed into the layer below. Each layer of the stack has a Dialog-Aware Self-Attention component and an Utterance Recurrence Component. The hidden state of the categorization token and the historical context are fed through a feed-forward neural network at the end of the last layer to produce the recognized emotion.
- Fusion method. Individual modalities were fused using Embracenet+, which is presented as an improvement of the Embracenet approach in [15]. The architecture involves three simple Embracenet models working to improve the modalities’ correlation learning as well as the final results. Each Embracenet model used has one more linear layer and a dropout layer, which hardens the model a bit to improve learning. Figure 13 shows the Embracenet+ architecture. In it, a linear layer of 32 neurons (D1,1), a dropout layer with 0.5 decay probability and another linear layer of 16 neurons (D1,2) compose each of the altered docking layers. Additionally, a weighted sum, whose output is a vector of *n* probabilities (n= number of emotion categories), and a concatenation, whose output is a vector of 3n (due to the number of modalities), are used as fusion techniques. Afterwards, another Embracenet receives three vectors of 16, *n* and 3n values (that work as modalities). These vectors are handled by docking layers of one linear layer of 16 neurons each ($d^{\left(k\right)}$), leading to an extra linear layer of *n* neurons, which outputs the final prediction.

All modalities were trained with a batch size of 32 samples per step and 20 epochs. All modalities were optimized using the Adam algorithm, with a learning rate of 0.0001. The implementation was based on PyTorch 2.0 (https://pytorch.org/, accessed on 27 May 2023). All models were trained on GPU, using an Nvidia GeForce RTX 3070 with 12 GB of VRAM.

Using the described framework, tests were performed on the IEMOCAP dataset and the results showed a 79% F1-Score and 77.6% accuracy for the fusion of the three modalities. On the individual modalities, i.e., Face-F, Audio-A and Text-T, the model obtained an average accuracy of 44%, 58.3% and 83.5%, respectively.

## 5. Dataset Evaluation

**Figure 14:**
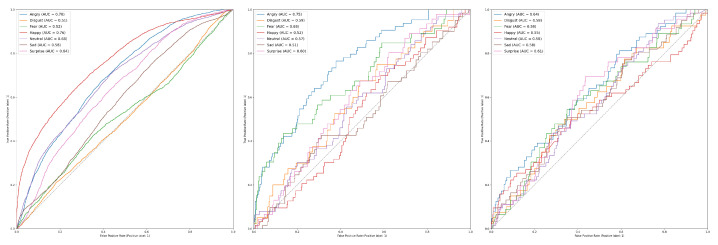
ROC Curves of emotions per modality on AFEW Dataset. Left to right: Face modality: happiness shows the highest Area Under the Curve (AUC) with 0.76; disgust performs the worst, with an AUC of 0.51. As disgust is a more complex facial expression, the model struggles to differentiate it from other expressions. Audio modality: anger shows an AUC of 0.75, while both sadness and happiness perform the worst with AUCs of 0.52 and 0.51, respectively. Text modality: anger has the highest AUC with 0.64. However, most emotions have very similar performance, with the lowest performing emotion being happiness with an AUC of 0.55.

Table 6 shows the results of our tests using the AFEW dataset. We achieved a mean accuracy of 26.91%,21.67%,22.19% on face, audio and text modalities, respectively. The best performing emotions on our test were happiness with 55.208% for faces, neutral with 33.333% for audio and 43.750% for anger in text mode. One of the main issues we noticed is how the architecture under-performs in certain categories, despite not having a high imbalance of samples for each emotion. disgust and surprise were the worst performers in each mode. AFEW is built with faces of actors who have more pronounced expressions and therefore should be more distinguishable. Figure 14 shows the ROC curves of the three modalities using AFEW. In the faces modality, the emotion happiness has an AUC of 0.61. While for audio and text, anger is the emotion with the highest AUC (0.75 and 0.68, respectively). This may be due to the fact that the audio of the set presents more detailed voice tonalities, coming from professional actors.

**Figure 15:**
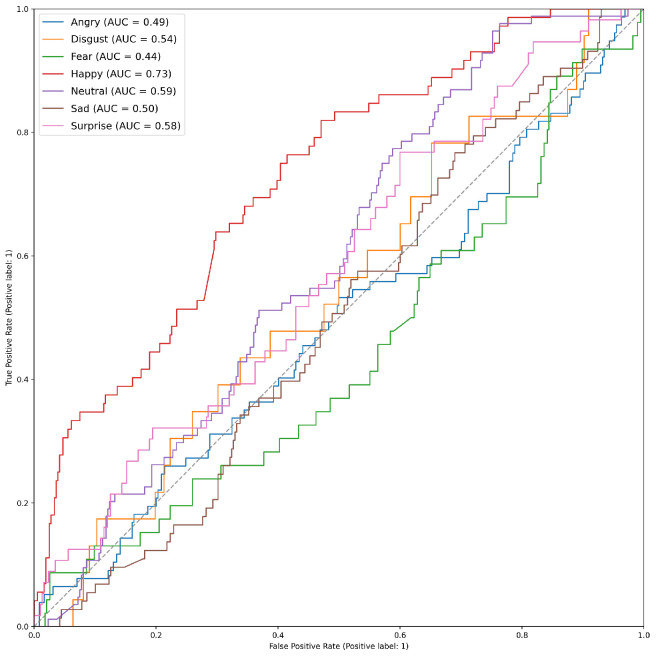
ROC Curve of SFEW dataset. Happiness has the highest AUC with 0.73, while the worst performing emotion is fear with an AUC of 0.44.

Table 7 shows the results for the SFEW dataset. We achieved a mean accuracy of 21.81%, with happiness and anger being most correctly classified, with accuracies of 38.889% and 36.364%, respectively. Similarly to AFEW, which is an expansion of SFEW, it performs badly with surprise and disgust, while having no detection for fear. Figure 15 presents the ROC curve of the SFEW face modality. The emotion with the highest AUC is happiness with 0.72.

**Figure 16:**
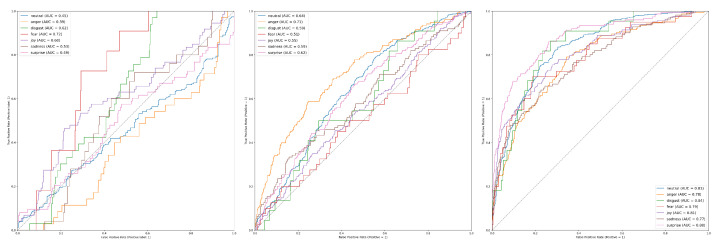
ROC Curves of emotions per modality for the MELD Dataset. Left to right: Face modality: the highest performing emotion is fear, with an AUC of 0.72. However, both neutral and anger have an AUC lower that 0.5 (0.45 and 0.39, respectively), thus confusing most of these examples with other emotions. Audio modality: anger has an AUC of 0.71 and both neutral (AUC=0.64) and surprise (AUC=0.62) have more favorable results. Fear performs the worst with an AUC of 0.51. Text modality: the best performing emotion is surprise with an AUC of 0.88, while the worst is sadness with 0.77. This high performance is expected as this dataset was designed considering text classifications.

Table 8 shows the results on the MELD dataset. Mean F1s were achieved of 15.9%, 34.6% and 53.7% for face, audio and text, respectively. Figure 16 shows the ROC curve for these results. The modality that exhibited the best performance is text mode with a mean accuracy of 57.40%, which is congruent with the fact that this dataset was designed for emotion detection in conversations. The face modality presents the worst results. This is due to the difficulty of building a new dataset for images and of relabeling them. Some of the audio clips are cut or missing the first syllable or might have extra sounds at the beginning or end. Other audio clips sound unnatural. Even though the texts are complete in the CSV file, rebuilding them from the audio files is not possible due to this problem.

**Figure 17:**
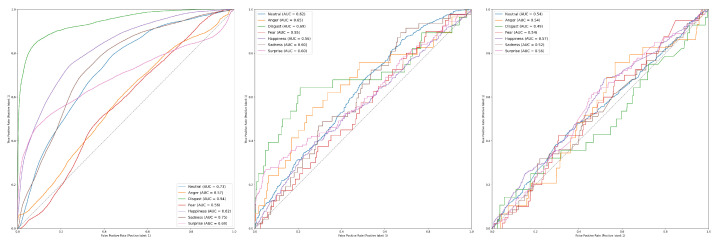
ROC Curves of emotions per modality on AffWild2 Dataset. Left to right: Face modality: the best performing emotion was disgust with an AUC of 0.94, while the lowest was fear with an AUC of 0.56. This modality performs well in comparison with the other modalities, as AffWild2 is designed for face recognition. Audio modality: the best and worst performing emotions were, respectively, disgust (AUC=0.69) and fear (AUC=0.69). Text modality: the performance of this modality was poor due to the limitations of the AffWild2 Dataset; happiness achieved an AUC of 0.57, while disgust scored lowest with an AUC of 0.49.

Table 9 shows the results on the AffWild2 dataset. The VGG19 architecture using this dataset achieved a weighted F1-Score of 41% on its original face modality. However, since the dataset was not designed for other modalities, such as audio or text, performance was low in these modalities (F1-Scores of 32% in audio and 29% in text). One of the main reasons for this is the source of the speech (if produced). A significant portion of the dataset is composed of reaction videos to entertainment content. As such, the sound in the emotion segment might not coincide with the labeled emotion presented as a label, since these were annotated with faces in mind. Figure 17 shows the ROC curves for each emotion for each modality. Disgust presents the highest area under the curve in both faces and audio (0.94 and 0.69, respectively). While in the text modality, happiness presents the highest AUC with 0.57.

Finally, we evaluated all three modalities using the Embracenet+ fusion method. Table 10 shows the accuracy results using all modalities. AffWild2 performed the best with a weighted accuracy of 58.64%, while AFEW has a weighted accuracy of 18.87 and MELD has an accuracy of 45.63. In the case of AffWild2, both face and text show good results, both individually and fused. The audio modality has the lowest performance, as the audio features are limited by the source data. For the AFEW dataset, the best performance was achieved by the facial modality by itself, with 19.68%. The performance seems on par with what was expected from the unimodal experiment. With MELD, only text modality performed as expected, due to, as was mentioned, this dataset being built for text and audio. However, the combination of audio and text achieved an accuracy of 20.39%. The results obtained might indicate some problems with our features; the face detector by itself has weak performance, as seen in Table 8. In comparison with similar models for each dataset, our model performs similarly or better. However, there were significant differences in how these models were evaluated, compared to ours. For example, for AffWild2, Zhang et al. [66] reported a multimodal accuracy of 39.4%, but they added the emotional category “Other”, which we did not evaluate and these cases are a highly represented class inside AffWild2. The work of Li et al. [46] on AFEW performs better on audio and face tasks compared to us, because they used a combination of more complex architectures than we used for the same task. Finally, on the MELD dataset, we achieved similar results to Hu et al. [55].

## 6. Discussion

The literature review and the large number of papers that describe models under development show the interest in the area of ER and the challenges still present. However, there is still no dataset or database designed and oriented entirely for multimodal ER. We can find datasets designed in particular for face recognition but not for the other modalities. In this case there are situations where the audio and text do not correspond to the facial expression, imposing the need to re-label the data for these modalities. In other situations where the focus of the dataset is oriented to text recognition in conversations, the facial modality takes a back seat, imposing the need for additional preprocessing. In the case of raw video data there are group images where multiple faces are identified. This implies the need to first perform face recognition and cropping, with the associated difficulty of assigning the audio to the corresponding person.

Most of the datasets do not have a good class balance, so the models generated from them have an important bias present, impacting the classification by overfitting the model to one class or another. As a result, metrics with accuracy are not the most appropriate for indicating good performance of the models. Many of these models have the problem that their generalization capacity is compromised because they are not able to predict correctly when faced with new cases not seen during the training process. Although there are multiple methods to balance the classes, e.g., by discarding samples or by generating synthetic samples (oversampling), the main limitation relates to the frequency of recording of these emotions. Certain expressions have a different duration, in accordance with the context, in addition to the differences of gesticulation within the same emotion. Therefore, a greater diversity of examples is needed so that the model can improve its classification capacity.

AFEW and SFEW are based on images with actors who gesticulate markedly to express their emotions, which could compromise their adaptation to reality. Due to its vastness and variety of emotions, the AffWild2 dataset improved the performance of all networks trained with it compared to networks trained with other datasets. However, its focus is facial, leaving inconsistencies in both audio and text modalities. In fact, because this dataset is labeled with faces in mind, emotion responses could differ depending on what is happening in context. Another issue is the mean length of each utterance, as most labeled samples are too short to enunciate a complete word. This might create bias towards short voice expressions of emotion, such as a scream or a laugh.

In AffWild2, most of the videos provided are of reactionary nature towards multimedia content or from interactions with other people off-camera; the labeled emotion could be different to what is happening in audio. For example, a child might be shown smiling, but in the background the mother is talking about her problems. As such, particular phrases extracted from the audio might have opposing emotions, increasing confusion in the model and lowering performance. The solution to this issue is simple yet costly, requiring manual labeling of the audio emotion in context.

In AFEW and AffWild2, the transcription of the audio by the same set is missing, limiting the performance of the text model. This is because it is dependent on the ability of the automatic transcription system and on its performance in in-the-wild environments. Thus, the text may not correspond to what was actually uttered by the subject. On the other hand, the MELD dataset was conceived for ER in conversations so the facial modality requires more preprocessing. Most videos include more than one person and therefore the images we can extract from them require first a separation and cropping of faces in addition to the corresponding labeling.

Some audio items were discarded from our test due to low quality sources and data corruption on provided files. This impacted the number of samples actually available for training. Other issues present with audio items are related to the issue of source quality. Since some videos, particularly in AffWild2, were extracted from Internet and social media platforms, some of them correspond to people reacting to Internet content or other videos, films, etc. As a result, most of the time the audio background does not match the perceived emotion present on video. Another example of these situations is videos of people interacting with other people off-camera, which increases difficulty of emotion recognition associated with these videos.

The text modality is generally missing a required automatic transcription from audios (except for MELD). Manual transcription is not cost-effective due to the number and length of the videos, as well as the range of accents present in the dataset.

Our multimodal strategy using the Embracenet+ Architecture indicates that multiple information items from different sources can be useful to improve emotion recognition, in comparison with the unimodal strategy. However, some of the results are contradictory to what was expected. For example, when using the MELD dataset, which was designed for audio and text emotion recognition, we expected good performance using those modalities, but reached a weighted accuracy of 20.39%. Nevertheless, audio and text on the multimodal test reached 45.63% and 46.6%, respectively, and 43.14% and 57.4% as a unimodal model.

Nevertheless, the datasets achieve results consistent with the objective for which they were designed. In the case of AFEW, the model performed better in the face modality than AffWild2. The considerable number of samples in AffWild2 favors performance in the face modality. In the case of MELD, it outperforms the other datasets in text.

A possible solution to these problems seems to be the combination of multiple datasets. This would allow for a greater diversity of samples in different contexts, both in acted scenes and in day-to-day living situations. Improvements in the testing datasets regarding modalities other than images could improve performance on in-the-wild emotion recognition tasks, including a correct labeling of the different data sources.

The proposed approach is a light and not so complex ensemble of models; therefore, the resource requirements for both training and deployments are lower. Initially, the framework of Heredia et al. was designed to be executed in a human–robot interaction environment [15]. The restrictions in this environment precisely favor less complex and lighter models. However, the framework was trained on the Lab dataset IEMOCAP which has very different characteristics to in-the-wild datasets reported in this work. Even though this framework has to be optimized and new components have to be tested, the framework establishes a starting point and allows us to make an evaluation on the critical conditions presented by in-the-wild datasets. Additionally, the results show that extra preprocessing tasks have to be carried out in order to yield better performance.

## 7. Conclusions

There are a variety of datasets that have been designed for emotion recognition. In this work we have evaluated ER performance using a previously designed architecture and four in-the-wild datasets: AFEW, SFEW, MELD and AffWild2. In the literature, it is common to find architectures based on Deep Learning for emotion recognition. We use an ensemble of pre-trained networks and some performance metrics such as accuracy and F1-Score. The results show that our models can effectively identify emotions using cropped images, audios and transcriptions of what is being said. However, available datasets have not been designed for multimodal ER tasks.

Comparing the results obtained with the studied datasets, for face modality our best performing dataset was AffWild2 with a mean accuracy of 53.98% and a mean F1-Score of 0.514. This is mostly because of the large number of available image samples provided in the dataset, allowing better generalization. For audio modality, our model performed best with the AffWild2 dataset with a mean accuracy of 46.93% and an F1-Score of 0.473, somewhat better than MELD, which followed with a mean accuracy of 43.14% and a mean F1 of 0.356. Though MELD is a dataset that was designed for audio and text modalities, the number of available examples per class is somewhat unbalanced, especially in less available classes such as disgust or fear, which is understandable since the original source is a comedy show. Within the text modality, the best mean performance was achieved with AffWild2, with an accuracy of 60.69%, but this result is skewed since for this dataset our model over-fitted due to the huge number of neutral and happiness class samples over the other classes. The best dataset for this task overall was MELD, with a mean accuracy of 57.40% and an F1-Score of 0.537. Since this dataset was designed for this particular task and included transcripted text of the sampled dialog, our model was able to classify with higher certainty compared with AffWild2 and AFEW. Transcriptions for these two datasets were dependant on both stored sound quality and the speech recognition model used.

When comparing all three datasets for multimodal fusion, AffWild2 had the best performance overall, despite having more data availability biases. This could be further improved with more labeled data, especially from different sources and environments and data augmentation for the least represented examples.

The next steps in this research include the configuration of a new dataset from the existing in-the-wild datasets, improving the preprocessing of each data source and its labeling for multimodal tasks and retraining on our set of networks after incorporating some optimization techniques with the goal of achieving unimodal and multimodal performance.

## Figures and Tables

**Table 1:** Related works.

Author	Method	Dataset/Database Name	Details	Accuracy
Riaz et al. [43]	Convolutional Neural Network (eXnet)	FER-2013 CK+ RAF-DB	12 Convolution Batch- normalization ReLU + 2 Fully connected layers	71.67% 95.63% 84%
Chen et al. [41]	Chen’s method	HEU-part1 HEU-part2 CK+ AFEW 7.0	CNN + GRU + 3D ResNeXt + OpenSMILE + MMA	49.22% (HEU-part1) 55.04% (HEU-part2)
Samadiani et al. [36]	Random Forest (RF)	AFEW 4.0	Generalized Procrustes analysis + RF	39.71%
Huet al. [45]	Deep CNNs with top-layer supervision (SSE)	AFEW 7.0	Fusion of 1 SSE-ResNet + 1 SSE-DenseNet + 1 SSE-HoloNet + 1 hand-crafted model + 1 audio model	60.34%
Li et al. [46]	Bimodality fusion method	AFEW 7.0 AffectNet	VGG-Face, BLSTM, ResNet-18, DenseNet-121, VGG-AffectNet	62.8%
Salah et al. [47]	Salah’s method	AFEW 5.0 AFEW 7.0 FER-2013	VGG-Face + Kernel Extreme Learning Machine + Partial Least Squares regression	54.55% (AFEW 5.0) 52.11% (AFEW 7.0)
Yu and Zhang [48]	CNN, Relu, the log likelihood loss, the hinge loss	FER-2013- SFEW 2.0	5 convolutional layers, 3 stochastic pooling layers and 3 fully connected layers. JDA, DCNN, MoT detectors	52.29% (validation) 58.06% (test)
Munir et al. [49]	SMO, KNN, simple logistic MLP	SFEW	Fast Fourier transform and contrast limited adaptive histogram equalisation and merged binary pattern code	96.2% (holistic based) 65.7% (division based)
Cai et al. [50]	CNN + IL	CK+, MMI, Oulu-CASIA, SFEW	3 convolutional layers, full connected layers with an IL and the Softmax loss at the decision layer	SFEW results: 51.83% (validation) 56.99% (test)
Ruan et al. [51]	ResNet-18, full connected layers	CK+, MMI, Oulu-CASIA, RAF-DB, SFEW	Ensemble of networks (FDN, FRN)	89.47% (RAF-DB) 62.16% (SFEW)
Xie et al. [53]	Transformer-based crossmodality fusion with the Embracenet architecture	MELD	GPT, WaveRNN, FaceNet + GRU, transformer-based fusion mechanism with Embracenet	64%
Ho et al. [54]	Multi-Level Multi-Head Fusion Attention mechanism and recurrent neural network (RNN)	IEMOCAP, MELD, CMU-MOSEI	MFCC, BERT, multi-head attention technique	MELD results: 63.26% (accuracy), 60.59% (F1-Score)
Huet al. [55]	Multimodal fused graph convolutional network (MMGCN)	IEMOCAP, MELD	Modality Encoder (full connected networks + LSTM), Multimodal Graph Convolutional Network, Emotion Classifier (full connected network)	66.22% (IEMOCAP) and 58.65% (MELD)
Barros and Sciutti [58]	FaceChannelS	AffWild2	VGG16 base with fewer parameters and LSTM for temporal information.	34% Accuracy by frame. 41% as sequences.
Liu et al. [59]	Multi-Task Assisted Correction (MTAC)	AffWild2, RAF-DB, AffectNet	ResNet-18, DenseNet	62.78% ResNet18. 63.51% DenseNet
Yu et al. [63]	Ensemble of models and multi-fold training	AffWild2	ResNet50, EfficientNet, InceptionNet.	F1-Score ResNet50 0.263, EfficientNet 0.296, InceptionNet 0.27
Zhang et al. [66]	Unified transformer-based multimodal framework	AffWild2	3 GRU + MLP	F1-Score 39.4% (official fold AffWild2)
This work	Adaptive multimodal framework	AFEW, SFEW, MELD, AffWild2	VGG19, ResNet50, DialogXL, Embracenet+	18.87% (AFEW), 21.81% (SFEW), 45.63% (MELD), 58.94% (AffWild2)

**Table 2:** Datasets.

Acronym	Dataset/Database Name	Environment	Modality	Samples	Emotions
AffectNet [67]	Facial Affect from the InterNet	Wild	Facial	456,000 images	80,276 Neutral, 146,198 Happy, 29,487 Sad, 16,288 Surprise, 8191 Fear, 5264 Disgust, 28,130 Anger, 5135 Contempt, 35,322 None, 13,163 Uncertain, 88,895 Non-Face
AFEW 4.0 [68]	Acted Facial Expression in the Wild	Wild	Facial, audio, and posture	1426 video clips	194 Anger, 123 Disgust, 156 Fear, 165 Sadness, 387 Happiness, 257 Neutral, 144 Surprise
AFEW 5.0 [69]	Acted Facial Expression in the Wild	Wild	Facial, audio, and posture	1645 video clips	Angry, Disgust, Fear, Sadness, Happiness, Neutral, Surprise
AFEW 7.0 [70]	Acted Facial Expression in the Wild	Wild	Facial, audio, and posture	1809 video clips	295 Angry, 154 Disgust, 197 Fear, 258 Sadness, 357 Happiness, 400 Neutral, 148 Surprise
AffWild2 [57]	AffWild2 database	Wild	Facial	558 Videos with 2.8 M frames	22,699 Anger, 16,067 Disgust, 17,488 Fear, 129,974 Happiness, 259,456 Neutral, 272,310 Other, 103,908 Sadness, 43,947 Surprise
CK+ [71]	Extended Cohn- Kanade Dataset (CK+)	Lab	Facial	327 labeled facial videos	45 Angry, 18 Contempt, 59 Disgust, 25 Fear, 69 Happy, 28 Sadness, 83 Surprise
FER-2013 [72]	Facial Expression Recognition 2013 (FER-2013)	Wild	Facial	35,887 images	4953 Anger, 547 Disgust, 5121 Fear, 8989 Happiness, 6077 Sadness, 4002 Surprise, 6198 Neutral
HEU-part1 [41]	Multimodal emotion	Wild	Face and posture	16,569 videos	1631 Anger, 691 Bored, 1662 Confused, 1097 Disappointed, 881 Disgust, 1268 Fear, 4313 Happy, 2568 Neutral, 1529 Sad, 929 Surprise
HEU-part2 [41]	Multimodal emotion	Wild	Face, speech and posture	2435 videos	389 Anger, 50 Bored, 131 Confused, 108 Disappointed, 162 Disgust, 221 Fear, 460 Happy, 350 Neutral, 364 Sad, 243 Surprise
SFEW 2.0 [73]	Static Facial Expression in Wild	Wild	Facial	1322 samples	255 anger, 75 disgust, 124 fear, 228 neutral, 256 happiness, 234 sadness and 150 surprise
RAF-DB [74]	Real-world Affective Faces Database (RAF-DB)	Wild	Facial	30,000 facial images	1619 Surprised, 355 Fearful, 877 Disgusted, 5957 Happy, 2460 Sad, 867 Angry, 560 Fearfully surprised, 148 Disgustedly surprised, 697 Happily surprised, 86 Sadly surprised, 176 Angrily surprised, 129 Sadly fearful, 8 Fearfully disgusted, 266 Happily disgusted, 738 Sadly disgusted, 841 Angrily disgusted, 150 Fearfully angry, 163 Sadly angry
MELD [52]	Multimodal EmotionLines Dataset	Wild	Audio, visual, and textual	13,158 videos	1607 anger, 361 disgust, 1002 sadness, 2308 joy, 6436 neutral, 1636 surprise, 358 fear
CMU-MOSEI [75]	CMU Multimodal Opinion Sentiment and Emotion Intensity	Wild	Language, vision, and audio	23,453 annotated sentences from more than 1000 online speakers and 250 different topics	1438 happy, 502 sad, 384 angry, 55 fear, 150 disgust, 78 surprise
IEMOCAP [76]	Interactive emotional dyadic motion capture database	Lab	Audio, video and facial	10,038 samples	1229 anger, 1182 sadness, 495 happiness, 575 neutral, 2505 excited, 24 surprise, 135 fear, 4 disgust, 3830 frustration, 59 other

**Table 3:** Number of images available in SFEW per emotion.

Emotion	Train	Validation	Total
Angry	178	77	255
Disgust	52	23	75
Fear	78	46	124
Happy	184	72	256
Neutral	144	84	228
Sad	161	73	234
Surprise	94	56	150

**Table 4:** Images per emotion in AffWild2.

Emotion	Train	Validation	Total
Anger	16,573	6126	22,699
Disgust	10,771	5296	16,067
Fear	9080	8408	17,488
Happiness	95,463	34,511	129,974
Neutral	177,198	82,258	259,456
Other	165,866	106,444	272,310
Sadness	78,751	25,157	103,908
Surprise	31,615	12,332	43,947

**Table 5:** In-the-wild datasets.

Dataset	Samples	Modalities	# Emotions	Size
AFEW	1154 faces, 1154 audios, 747 texts	Face, Audio, Text	7	2.65 GB
SFEW	1007 faces	Face	7	995 MB
MELD	7487 audios, 7487 texts	Face, Audio, Text	7	10.1 GB
AffWild2	865,849 images extracted from 548 videos	Face	8	17.5 GB

**Table 6:** AFEW multimodal evaluation metrics.

Emotion	Face			Audio			Text		
	Acc.	F1-Score	Support	Acc.	F1-Score	Support	Acc.	F1-Score	Support
Angry	27.05	0.30	3216	31.25	0.38	64	43.75	0.36	64
Disgust	8.86	0.10	2404	17.50	0.17	40	2.50	0.04	40
Fear	14.07	0.13	1805	28.26	0.27	46	15.22	0.19	46
Happy	55.21	0.43	3389	22.22	0.18	63	30.16	0.22	63
Neutral	37.05	0.37	3614	33.33	0.26	63	34.92	0.26	63
Sad	18.19	0.19	3287	6.55	0.09	61	11.48	0.15	61
Surprise	9.16	0.11	2130	8.70	0.11	46	2.17	0.04	46
Weighted Average	26.91	0.26	19845	21.67	0.21	383	22.19	0.20	383

**Table 7:** SFEW Unimodal evaluation metrics.

Emotion Category	Face		
	Accuracy	F1-Score	Support
Angry	36.36	0.23	77
Disgust	0.00	0.00	23
Fear	0.00	0.00	46
Happy	38.89	0.37	72
Neutral	19.05	0.20	84
Sad	24.66	0.22	73
Surprise	7.14	0.11	56
Weighted Average	21.81	0.19	431

**Table 8:** MELD unimodal evaluation metrics.

Emotion	Face			Audio			Text		
	Acc.	F1-Score	Support	Acc.	F1-Score	Support	Acc.	F1-Score	Support
Neutral	10.91	0.18	229	80.17	0.62	469	86.14	0.72	469
Anger	11.42	0.09	35	45.10	0.36	153	28.76	0.34	153
Disgust	36.36	0.16	33	0.00	0.00	22	13.64	0.22	22
Fear	18.18	0.06	11	0.00	0.00	40	2.50	0.04	40
Happiness	15.06	0.18	73	0.61	0.00	163	48.47	0.51	163
Sadness	4.00	0.02	25	0.90	0.02	111	21.62	0.31	111
Surprise	15.06	0.17	73	20.67	0.23	150	54.00	0.56	150
Weighted average	13.77	0.16	479	43.14	0.35	1108	57.40	0.54	1108

**Table 9:** Results of each modality using AffWild2 Dataset.

Emotion	Face			Audio			Text		
	Acc.	F1-Score	Support	Acc.	F1-Score	Support	Acc.	F1-Score	Support
Neutral	74.34	0.70	82,258	60.20	0.63	897	96.99	0.75	897
Anger	6.25	0.09	6126	3.44	0.40	29	0.0	0.0	29
Disgust	59.42	0.62	5296	7.14	0.10	28	0.0	0.0	28
Fear	2.57	0.03	8408	0.0	0.00	40	0.0	0.0	40
Happiness	60.43	0.55	34,511	38.87	0.30	301	6.98	0.12	301
Sadness	12.53	0.195	25,157	4.26	0.045	47	0.0	0.0	47
Surprise	40.87	0.32	12,332	21.43	0.23	126	0.0	0.0	126
Weighted Average	53.98	0.514	174,088	46.93	0.473	1468	60.69	0.484	1468

**Table 10:** Ablation Study of each modality in Embracenet+ for AFEW, MELD and AffWild2 datasets considering the accuracy measures.

Modalities			Evaluated Datasets					
			AFEW		MELD		AffWild2	
**Face**	**Audio**	**Text**	**Li et al. [46]**	**Ours**	**MMGCN [55]**	**Ours**	**Fusion Transformer [66]**	**Ours**
x			53.91%	19.58%	33.27%	45.63%	-	62.01%
	x		25.59%	16.17%	42.63%	45.63%	-	40.52%
		x	-	14.82%	57.72%	46.60%	-	61.05%
x	x		54.30%	18.06%	-	45.63%	32.60%	48.91%
x		x	-	18.87%	57.92%	45.63%	36.30%	60.78%
	x	x	-	15.90%	58.02%	20.39%	51.20%	52.52%
x	x	x	-	18.87%	58.65%	45.63%	39.40%	58.94%

## Data Availability

Not applicable.

## References

[1] Dzedzickis A., Kaklauskas A., Bucinskas V. (2020). Human Emotion Recognition: Review of Sensors and Methods. Sensors.

[2] Wang Z., Ho S.B., Cambria E. (2020). A Review of Emotion Sensing: Categorization Models and Algorithms. Multimed. Tools Appl..

[3] Shaver P., Schwartz J., Kirson D., O’Connor C. (1987). Emotion Knowledge: Further Exploration of a Prototype Approach. J. Pers. Soc. Psychol..

[4] Ekman P. (1992). An Argument for Basic Emotions. Cognit. Emo.

[5] Stahelski A., Anderson A., Browitt N., Radeke M. (2021). Facial Expressions and Emotion Labels Are Separate Initiators of Trait Inferences from the Face. Front. Psychol..

[6] Schulz A., Thanh T.D., Paulheim H., Schweizer I. A Fine-Grained Sentiment Analysis Approach for Detecting Crisis Related Microposts. Proceedings of the 10th International ISCRAM Conference.

[7] Latinjak A. (2012). The Underlying Structure of Emotions: A Tri-Dimensional Model of Core Affect and Emotion Concepts for Sports. Rev. Iberoam. Psicol. Ejecicio Deporte (Iberoam. J. Exerc. Sport Psychol.).

[8] Feng K., Chaspari T. (2020). A Review of Generalizable Transfer Learning in Automatic Emotion Recognition. Front. Comput. Sci..

[9] Lhommet M., Marsella S.C., Calvo R.A., D’Mello S.K., Gratch J., Kappas A. (2014). Expressing Emotion through Posture and Gesture. Oxford Handbook of Affective Computing.

[10] Pease A., Chandler J. (1997). Body Language.

[11] Cowen A.S., Keltner D. (2020). What the Face Displays: Mapping 28 Emotions Conveyed by Naturalistic Expression. Am. Psychol..

[12] Mittal T., Guhan P., Bhattacharya U., Chandra R., Bera A., Manocha D. EmotiCon: Context-Aware Multimodal Emotion Recognition Using Frege’s Principle. Proceedings of the 2020 IEEE/CVF Conference on Computer Vision and Pattern Recognition (CVPR).

[13] Mittal T., Bhattacharya U., Chandra R., Bera A., Manocha D. (2020). M3ER: Multiplicative Multimodal Emotion Recognition using Facial, Textual and Speech Cues. Proc. Aaai Conf. Artif. Intell. AAAI.

[14] Subramanian G., Cholendiran N., Prathyusha K., Balasubramanain N., Aravinth J. Multimodal Emotion Recognition Using Different Fusion Techniques. Proceedings of the 2021 Seventh International Conference on Bio Signals, Images and Instrumentation (ICBSII).

[15] Heredia J., Lopes-Silva E., Cardinale Y., Diaz-Amado J., Dongo I., Graterol W., Aguilera A. (2022). Adaptive Multimodal Emotion Detection Architecture for Social Robots. IEEE Access.

[16] Poria S., Chaturvedi I., Cambria E., Hussain A. Convolutional MKL Based Multimodal Emotion Recognition and Sentiment Analysis. Proceedings of the 2016 IEEE 16th International Conference on Data Mining (ICDM).

[17] Kratzwald B., Ilić S., Kraus M., Feuerriegel S., Prendinger H. (2018). Deep Learning for Affective Computing: Text-Based Emotion Recognition in Decision Support. Decis. Support. Syst..

[18] Soleymani M., Garcia D., Jou B., Schuller B., Chang S.F., Pantic M. (2017). A survey of Multimodal Sentiment Analysis. Image Vis. Comput..

[19] Ahmed N., Aghbari Z.A., Girija S. (2023). A systematic Survey on Multimodal Emotion Recognition using Learning Algorithms. Intell. Syst. Appl..

[20] Xu H., Zhang H., Han K., Wang Y., Peng Y., Li X. (2019). Learning Alignment for Multimodal Emotion Recognition from Speech. arXiv.

[21] Salama E.S., El-Khoribi R.A., Shoman M.E., Wahby Shalaby M.A. (2021). A 3D-convolutional Neural Network Framework with Ensemble Learning Techniques for Multi-Modal Emotion recognition. Egypt. Inform. J..

[22] Cimtay Y., Ekmekcioglu E., Caglar-Ozhan S. (2020). Cross-Subject Multimodal Emotion Recognition Based on Hybrid Fusion. IEEE Access.

[23] Tripathi S., Tripathi S., Beigi H. (2018). Multi-Modal Emotion Recognition on IEMOCAP Dataset using Deep Learning. arXiv.

[24] Li C., Bao Z., Li L., Zhao Z. (2020). Exploring Temporal Representations by Leveraging Attention-Based Bidirectional LSTM-RNNs for Multi-Modal Emotion Recognition. Inf. Process. Manag..

[25] Liu W., Qiu J.L., Zheng W.L., Lu B.L. (2021). Comparing Recognition Performance and Robustness of Multimodal Deep Learning Models for Multimodal Emotion Recognition. IEEE Trans. Cogn. Develop. Syst..

[26] Ranganathan H., Chakraborty S., Panchanathan S. Multimodal Emotion Recognition Using Deep Learning Architectures. Proceedings of the 2016 IEEE Winter Conference on Applications of Computer Vision (WACV).

[27] Abdullah S.M.S.A., Ameen S.Y.A., Sadeeq M.A., Zeebaree S. (2021). Multimodal Emotion Recognition Using Deep Learning. J. Appl. Sci. Technol. Trends..

[28] Tzirakis P., Trigeorgis G., Nicolaou M.A., Schuller B.W., Zafeiriou S. (2017). End-to-End Multimodal Emotion Recognition Using Deep Neural Networks. IEEE J. Sel. Top. Signal Process..

[29] Alaba S.Y., Nabi M.M., Shah C., Prior J., Campbell M.D., Wallace F., Ball J.E., Moorhead R. (2022). Class-Aware Fish Species Recognition Using Deep Learning for an Imbalanced Dataset. Sensors.

[30] Zhao M., Liu Q., Jha A., Deng R., Yao T., Mahadevan-Jansen A., Tyska M.J., Millis B.A., Huo Y. (2021). VoxelEmbed: 3D Instance Segmentation and Tracking with Voxel Embedding based Deep Learning. Machine Learning in Medical Imaging.

[31] Jin B., Cruz L., Gonçalves N. (2022). Pseudo RGB-D Face Recognition. IEEE Sens. J..

[32] Yao T., Qu C., Liu Q., Deng R., Tian Y., Xu J., Jha A., Bao S., Zhao M., Fogo A.B. (2021). Compound Figure Separation of Biomedical Images with Side Loss. Proceedings of the Deep Generative Models and Data Augmentation, Labelling and Imperfections: First Workshop, DGM4MICCAI 2021 and First Workshop, DALI 2021, Held in Conjunction with MICCAI 2021.

[33] Jin B., Cruz L., Gonçalves N. (2020). Deep Facial Diagnosis: Deep Transfer Learning from Face Recognition to Facial Diagnosis. IEEE Access.

[34] Zheng Q., Zhao P., Li Y., Wang H., Yang Y. (2020). Spectrum Interference-based Two-Level data Augmentation Method in Deep Learning for Automatic Modulation Classification. Neural Comput. Appl..

[35] Garcia-Garcia J.M., Lozano M.D., Penichet V.M.R., Law E.L.C. (2023). Building a Three-Level Multimodal Emotion Recognition Framework. Multimed. Tools Appl..

[36] Samadiani N., Huang G., Luo W., Shu Y., Wang R., Kocaturk T. (2020). A Novel Video Emotion Recognition System in the Wild Using a Random Forest Classifier. Data Science.

[37] Samadiani N., Huang G., Luo W., Shu Y., Wang R., Kocaturk T. (2022). A multiple Feature Fusion Framework for Video Emotion Recognition in the Wild. Concurr. Computat. Pract. Exper..

[38] Liu T., Wang J., Yang B., Wang X. (2021). Facial Expression Recognition Method with Multi-Label Distribution Learning for Non-Verbal Behavior Understanding in the Classroom. Infrared Phys. Technol..

[39] Li X., Li T., Li S., Tian B., Ju J., Liu T., Liu H. (2023). Learning Fusion Feature Representation for Garbage Image Classification Model in Human–Robot Interaction. Infrared Phys. Technol..

[40] Kollias D., Zafeiriou S. (2019). Exploiting Multi-CNN Features in CNN-RNN based Dimensional Emotion Recognition on the OMG in-the-Wild Dataset. arXiv.

[41] Chen J., Wang C., Wang K., Yin C., Zhao C., Xu T., Zhang X., Huang Z., Liu M., Yang T. (2021). HEU Emotion: A Large-Scale Database for Multimodal Emotion Recognition in the Wild. Neural Comput. Applic..

[42] Li S., Deng W. (2020). Deep Facial Expression Recognition: A Survey. IEEE Trans. Affect. Comput..

[43] Riaz M.N., Shen Y., Sohail M., Guo M. (2020). eXnet: An Efficient Approach for Emotion Recognition in the Wild. Sensors.

[44] Dhall A., Sharma G., Goecke R., Gedeon T. (2020). EmotiW 2020: Driver Gaze, Group Emotion, Student Engagement and Physiological Signal based Challenges. Proceedings of the ICMI ’20: 2020 International Conference on Multimodal Interaction.

[45] Hu P., Cai D., Wang S., Yao A., Chen Y. (2017). Learning Supervised Scoring Ensemble for Emotion recognition in the wild. Proceedings of the ICMI’17: 19th ACM International Conference on Multimodal Interaction.

[46] Li S., Zheng W., Zong Y., Lu C., Tang C., Jiang X., Liu J., Xia W. (2019). Bi-modality Fusion for Emotion Recognition in the Wild. Proceedings of the ICMI’19: 2019 International Conference on Multimodal Interaction.

[47] Salah A.A., Kaya H., Gürpınar F. (2019). Video-Based Emotion Recognition in the Wild. Multimodal Behavior Analysis in the Wild.

[48] Yu Z., Zhang C. (2015). Image Based Static Facial Expression Recognition with Multiple Deep Network Learning. Proceedings of the 2015 ACM on International Conference on Multimodal Interaction.

[49] Munir A., Hussain A., Khan S.A., Nadeem M., Arshid S. (2018). Illumination Invariant Facial Expression Recognition using Selected Merged Binary Patterns for Real World Images. Optik.

[50] Cai J., Meng Z., Khan A.S., Li Z., O’Reilly J., Tong Y. Island Loss for Learning Discriminative Features in Facial Expression Recognition. Proceedings of the 2018 13th IEEE International Conference on Automatic Face & Gesture Recognition (FG 2018).

[51] Ruan D., Yan Y., Lai S., Chai Z., Shen C., Wang H. Feature Decomposition and Reconstruction Learning for Effective Facial Expression Recognition. Proceedings of the IEEE/CVF Conference on Computer Vision and Pattern Recognition (CVPR).

[52] Poria S., Hazarika D., Majumder N., Naik G., Cambria E., Mihalcea R. (2019). MELD: A Multimodal Multi-Party Dataset for Emotion Recognition in Conversations. Proceedings of the 57th Annual Meeting of the Association for Computational Linguistics.

[53] Xie B., Sidulova M., Park C.H. (2021). Robust Multimodal Emotion Recognition from Conversation with Transformer-Based Crossmodality Fusion. Sensors.

[54] Ho N.H., Yang H.J., Kim S.H., Lee G. (2020). Multimodal Approach of Speech Emotion Recognition Using Multi-Level Multi-Head Fusion Attention-Based Recurrent Neural Network. IEEE Access.

[55] Hu J., Liu Y., Zhao J., Jin Q. (2021). MMGCN: Multimodal Fusion via Deep Graph Convolution Network for Emotion Recognition in Conversation. arXiv.

[56] Kollias D., Tzirakis P., Nicolaou M.A., Papaioannou A., Zhao G., Schuller B., Kotsia I., Zafeiriou S. (2018). Deep Affect Prediction in-the-Wild: AffWild Database and Challenge, Deep Architectures and Beyond. arXiv.

[57] Kollias D., Zafeiriou S. (2019). Aff-Wild2: Extending the AffWild Database for Affect Recognition. arXiv.

[58] Barros P., Sciutti A. (2020). The FaceChannelS: Strike of the Sequences for the AffWild 2 Challenge. arXiv.

[59] Liu Y., Zhang X., Kauttonen J., Zhao G. (2022). Uncertain Facial Expression Recognition via Multi-task Assisted Correction. arXiv.

[60] He K., Zhang X., Ren S., Sun J. Deep Residual Learning for Image Recognition. Proceedings of the IEEE conference on computer vision and pattern recognition.

[61] Huang G., Liu Z., Van Der Maaten L., Weinberger K.Q. Densely Connected Convolutional Networks. Proceedings of the IEEE Conference on Computer Vision and Pattern Recognition.

[62] Guo Y., Zhang L., Hu Y., He X., Gao J. (2016). MS-Celeb-1M: A Dataset and Benchmark for Large-Scale Face Recognition. Proceedings of the Computer Vision–ECCV 2016: 14th European Conference.

[63] Yu J., Cai Z., He P., Xie G., Ling Q. (2022). Multi-Model Ensemble Learning Method for Human Expression Recognition. arXiv.

[64] Tan M., Le Q. (2019). Efficientnet: Rethinking Model Scaling for Convolutional Neural Networks. International Conference on Machine Learning.

[65] Szegedy C., Liu W., Jia Y., Sermanet P., Reed S., Anguelov D., Erhan D., Vanhoucke V., Rabinovich A. Going Deeper with Convolutions. Proceedings of the IEEE Conference on Computer Vision and Pattern Recognition.

[66] Zhang W., Qiu F., Wang S., Zeng H., Zhang Z., An R., Ma B., Ding Y. Transformer-based Multimodal Information Fusion for Facial Expression Analysis. Proceedings of the 2022 IEEE/CVF Conference on Computer Vision and Pattern Recognition Workshops (CVPRW).

[67] Mollahosseini A., Hasani B., Mahoor M.H. (2017). AffectNet: A Database for Facial Expression, Valence and Arousal Computing in the Wild. arXiv.

[68] Dhall A., Goecke R., Lucey S., Gedeon T. (2012). Collecting Large, Richly Annotated Facial-Expression Databases from Movies. IEEE Multimed..

[69] Dhall A., Ramana Murthy O.V., Goecke R., Joshi J., Gedeon T. (2015). Video and Image Based Emotion Recognition Challenges in the Wild: EmotiW 2015. Proceedings of the ICMI ’15: 2015 ACM on International Conference on Multimodal Interaction.

[70] Dhall A., Goecke R., Joshi J., Hoey J., Gedeon T. (2016). EmotiW 2016: Video and Group-Level Emotion Recognition Challenges. Proceedings of the ICMI ’16: 18th ACM International Conference on Multimodal Interaction.

[71] Lucey P., Cohn J.F., Kanade T., Saragih J., Ambadar Z., Matthews I. The Extended Cohn-Kanade Dataset (CK+): A Complete Dataset for Action Unit and Emotion-Specified Expression. Proceedings of the 2010 IEEE Computer Society Conference on Computer Vision and Pattern Recognition—Workshops.

[72] Goodfellow I.J., Erhan D., Carrier P.L., Courville A., Mirza M., Hamner B., Cukierski W., Tang Y., Thaler D., Lee D.H. (2013). Challenges in Representation Learning: A Report on Three Machine Learning Contests. arXiv.

[73] Dhall A., Goecke R., Lucey S., Gedeon T. Static Facial Expression Analysis in Tough Conditions: Data, Evaluation Protocol and Benchmark. Proceedings of the 2011 IEEE International Conference on Computer Vision Workshops (ICCV Workshops).

[74] Li S., Deng W., Du J. Reliable Crowdsourcing and Deep Locality-Preserving Learning for Expression Recognition in the Wild. Proceedings of the 2017 IEEE Conference on Computer Vision and Pattern Recognition (CVPR).

[75] Zadeh A., Liang P.P., Poria S., Cambria E., Morency L.P. (2018). Multimodal Language Analysis in the Wild: CMU-MOSEI Dataset and Interpretable Dynamic Fusion Graph. Proceedings of the Annual Meeting of the Association for Computational Linguistics.

[76] Busso C., Bulut M., Lee C.C., Kazemzadeh A., Mower E., Kim S., Chang J.N., Lee S., Narayanan S.S. (2008). IEMOCAP: Interactive Emotional Dyadic Motion Capture Database. Lang. Resour. Eval..

[77] Dhall A., Goecke R., Lucey S., Gedeon T. (2011). Acted Facial Expressions in the Wild Database. Technical Report TR-CS-11-02.

[78] Chen S.Y., Hsu C.C., Kuo C.C., Huang T.-H., Ku L.W. (2018). EmotionLines: An Emotion Corpus of Multi-Party Conversations. arXiv.

[79] Kollias D., Nicolaou M.A., Kotsia I., Zhao G., Zafeiriou S. Recognition of Affect in the Wild Using Deep Neural Networks. Proceedings of the 2017 IEEE Conference on Computer Vision and Pattern Recognition Workshops (CVPRW).

[80] Kollias D. Abaw: Valence-Arousal Estimation, Expression Recognition, Action Unit Detection & Multi-Task Learning Challenges. Proceedings of the IEEE/CVF Conference on Computer Vision and Pattern Recognition.

[81] Tomar S. (2006). Converting Video Formats with FFmpeg. Linux J..

[82] McFee B., Raffel C., Liang D., Ellis D.P., McVicar M., Battenberg E., Nieto O. Librosa: Audio and Music Signal Analysis in Python. Proceedings of the 14th Python in Science Conference.

[83] Lech M., Stolar M., Best C., Bolia R. (2020). Real-Time Speech Emotion Recognition Using a Pre-trained Image Classification Network: Effects of Bandwidth Reduction and Companding. Front. Comput. Sci..

[84] Ravanelli M., Parcollet T., Plantinga P., Rouhe A., Cornell S., Lugosch L., Subakan C., Dawalatabad N., Heba A., Zhong J. (2021). SpeechBrain: A General-Purpose Speech Toolkit. arXiv.

[85] Shen W., Chen J., Quan X., Xie Z. (2020). DialogXL: All-in-One XLNet for Multi-Party Conversation Emotion Recognition. arXiv.

[86] Simonyan K., Zisserman A. Very deep convolutional networks for large-scale image recognition. Proceedings of the 3rd International Conference on Learning Representations (ICLR 2015).

[87] Venkataramanan K., Rajamohan H.R. (2019). Emotion Recognition from Speech. arXiv.

